# Rofecoxib Attenuates the Pathogenesis of Amyotrophic Lateral Sclerosis by Alleviating Cyclooxygenase-2-Mediated Mechanisms

**DOI:** 10.3389/fnins.2020.00817

**Published:** 2020-08-13

**Authors:** Yan-Hui Zou, Pei-Pei Guan, Shen-Qing Zhang, Yan-Su Guo, Pu Wang

**Affiliations:** ^1^College of Life and Health Sciences, Northeastern University, Shenyang, China; ^2^Beijing Geriatric Healthcare Center, Xuanwu Hospital, Capital Medical University, Beijing, China

**Keywords:** cyclooxygenase-2, neuroinflammation, refecoxib, ALS, mechanism

## Abstract

Cyclooxygenase-2 (COX-2) is reported to be activated during the course of amyotrophic lateral sclerosis (ALS) development and progression. However, the roles of COX-2 in aggravating ALS and the underlying mechanism have been largely overlooked. To reveal the mechanisms, the canonical SOD1^G93A^ mouse model was used as an experimental model for ALS in the current study. In addition, a specific inhibitor of COX-2 activity, rofecoxib, was orally administered to SOD1^G93A^ mice. With this *in vivo* approach, we revealed that COX-2 proinflammatory signaling cascades were inhibited by rofecoxib in SOD1^G93A^ mice. Specifically, the protein levels of COX-2, interleukin (IL)-1β, and tumor necrosis factor (TNF)-α were elevated as a result of activation of astrocytes and microglia during the course of ALS development and progression. These proinflammatory reactions may contribute to the death of neurons by triggering the movement of astrocytes and microglia to neurons in the context of ALS. Treatment with rofecoxib alleviated this close association between glial cells and neurons and significantly decreased the density of inflammatory cells, which helped to restore the number of motor neurons in SOD1^G93A^ mice. Mechanistically, rofecoxib treatment decreased the expression of COX-2 and its downstream signaling targets, including IL-1β and TNF-α, by deactivating glial cells, which in turn ameliorated the progression of SOD1^G93A^ mice by postponing disease onset and modestly prolonging survival. Collectively, these results provide novel insights into the mechanisms of ALS and aid in the development of new drugs to improve the clinical treatment of ALS.

## Introduction

Amyotrophic lateral sclerosis (ALS) is a fatal progressive neurodegenerative disease (ND). ALS often occurs in adults. Clinically, 90% of patients have sporadic ALS (sALS) and 10% of patients have familial ALS (fALS). Patients with ALS usually die from respiratory failure 3–5 years after onset. Pathologically, ALS is characterized by the degeneration of upper and lower motor neurons. In addition to dyskinesia caused by muscle atrophy and muscle weakness, patients also experience dysphagia and dyspnea. At present, there is no effective treatment because the etiology of ALS remains unclear (Zufiria et al., 2016; Iannitti et al., 2018).

In 1993, an abnormal mutation of superoxide dismutase 1 (SOD1) was first identified as a key pathogenic factor of ALS (Rosen, 1993). Based on this observation, more than 30 different mutated pathogenic genes, including cyclooxygenase-2 (COX-2) (Drachman et al., 2002), tdp43 (TARDNA Binding Protein 43) (Neumann et al., 2006), Fus/TLS (Fused in Sarcoma) (Vance et al., 2009), and c9orf72 (Chromosome 9 Open Reading Frame 72) (DeJesus-Hernandez et al., 2011), were found to participate in regulating ALS over the next 20 years (Renton et al., 2014; Young et al., 2014). Research has shown that the damage to motor neurons caused by SOD1 mutation involves a variety of very complex pathophysiological processes (Ferraiuolo et al., 2011). This complexity has greatly hindered the progress of clinical treatment of ALS.

In recent years, the activation of COX-2 has been found to be closely related to the pathogenesis of ALS. Research has shown that COX-2 is highly activated in the spinal cords of SOD1^G93A^ mice, which are canonical ALS model mice (Almer et al., 2001). Treatment of SOD1^G93A^ mice with the COX-2 inhibitor, sc236 [a substance similar to celecoxib (CB)] obviously delays the development and progression of ALS by protecting motor neurons (Drachman et al., 2002). In addition, treatment with rofecoxib (a PTGS2 inhibitor) from postnatal day 60 has been found effective in delaying disease onset although not survival in SOD1^G93A^ mice (Azari et al., 2005). Further studies have shown that prostaglandin E_2_ (PGE_2_), a downstream metabolite of COX-2, can accelerate the development of ALS through its receptor EP2. Moreover, knockout of EP2 expression can significantly delay the course of ALS by prolonging survival in SOD1^G93A^ model mice (Liang et al., 2008). In contrast to EP2 activation, EP3 activation in SOD1^G93A^ mice significantly inhibits damage caused by PGE_2_ to motor neurons (Bilak et al., 2004). Therefore, COX-2 activation and metabolic dysfunction play important roles in the regulation of ALS.

As discussed above, it is generally believed that the role of COX-2 in promoting the development of ALS should not be ignored. Compared with the spinal cords of healthy people, those of ALS patients exhibit significantly greater COX-2 immune activity in activated microglia and macrophages (Yiangou et al., 2006). Although COX-2 is also expressed in non-glial cells (Maihofner et al., 2003), high expression of COX-2 in glial cells is likely to promote the development of ALS through an inflammatory mechanism (Almer et al., 2001). In addition, studies have confirmed that CD40 mediates the inflammatory mechanism of COX-2 to promote motor neuron loss (Okuno et al., 2004). Consistent with these observations, non-steroidal anti-inflammatory drugs (NSAIDs), which are inhibitors of COX-2, have been found to greatly alleviate the incidence of ALS in SOD1^G93A^ mice (Kiaei et al., 2005).

Although all this evidence highlights the potential contributions of COX-2 to the progression of ALS, the mechanisms have remained unknown. In this study, we found that rofecoxib can alleviate the associations between glial cells and neurons by decreasing the production of COX-2, IL-1β, and TNF-α in SOD1^G93A^ mice. The actions of rofecoxib in reducing neuroinflammation ameliorate the disease progression of SOD^G93A^ mice, providing a potential therapeutic strategy for ALS.

## Materials and Methods

### Reagents

The COX-2 inhibitor rofecoxib was obtained from Sigma-Aldrich (St. Louis, MO, United States). Antibodies specific for β-actin (rabbit, 1:5000), COX-2 (rabbit, 1:3000), NeuN (mouse, 1:5000), glial fibrillary acidic protein (GFAP) (rabbit, 1: 5000), IL-1β (rabbit, 1:4000), TNF-α (rabbit, 1:4000), Alexa Fluor-488 (1:400), Alexa Fluor-555 (1:400), and HRP-labeled secondary antibodies were purchased from Cell Signaling Technology (Danvers, MA, United States). DAPI was obtained from Beyotime Institute of Biotechnology (Haimen, Jiangsu, China). An Iba1 antibody (rabbit, 1:4000) was purchased from Wako Life Sciences (Wako, Tokyo, Japan). All reagents for sodium dodecyl sulfate polyacrylamide gel electrophoresis (SDS-PAGE) experiments were purchased from Bio-Rad Laboratories (Shanghai, China). All other reagents were from Invitrogen unless otherwise specified.

### Transgenic Mice and Treatments

C57BL/6J mice were purchased from Liaoning Changsheng Biotechnology Co., Ltd. (Benxi, Liaoning, China). SJL/J and B6SJL-Tg (SOD1^G93A^)/Cur/J mice were purchased from The Jackson Laboratory (Bar Harbor, ME, United States). Genotyping was performed 3–4 weeks after birth. In brief, 1 mm samples of mouse tails were placed in 150 μl of a solution of 50 mM NaOH and heated to 95°C for 30 min. The mixture was then cooled to room temperature and vortexed, and 12.5 μl of Tris–HCl was added to adjust the pH to 8.0. The extracted DNA was centrifuged at 1 × 10^4^ rpm for 2 min and 2 μl of supernatant was taken for PCR amplification. The forward and reverse primers for genotyping were 5′-CATCAGCCCTAATCCATCTGA-3′ and 5′-CGCGACTAACAATCAAAGTGA-3′, respectively. The forward and reverse primers for the internal control were 5′-CTAGGCCACAGAATTGAAAGATCT-3′ and 5′-GTAGGTGGAAATTCTAGCATCATCC-3′, respectively. The reaction mixtures were incubated at 95°C for 3 min and then subjected to 35 PCR cycles with the following temperature profile: 95°C for 45 s, 60°C for 30 s, and 72°C for 5 min. The DNA products of PCR were analyzed by electrophoresis on 2% agarose gels. Bands of 236 and 324 bp were expected for the positive mice and internal controls, respectively. Five mice per cage were housed in a controlled environment under standard room temperature, relative humidity, and a 12 h light/dark cycle with free access to food and water. According to the methods in a previous report (Niederberger et al., 2002), mice at the age of 1 month were orally administered rofecoxib (50 mg/kg/day) by gavage until the mice were unable to move from one side to the other in 30 s, which were considered as dead. In the control group, the mice were treated with the solvent vehicle. The general health and body weights of the animals were monitored every day. The onset and survival time of ALS was calculated and analyzed.

### Intragastric Administration

SOD1^G93A^ mice were intragastrically administered rofecoxib (50 mg/kg/day) by gavage. In brief, a suitable gavage needle was selected and fixed on a 1 ml syringe. The mice were then prepared via alignment of the head, neck, and body. The gavage needle was introduced into the corner of the mouth, pressed against the tongue, pushed inwardly against the upper jaw, and ultimately introduced into the esophagus. The injection volume was usually controlled to 0.1–0.2 ml/10 g. The gavage needle was removed gently after the injection.

### Gait Analysis

Gait analysis was used to determine the severity of motor weakness in the experimental mice. First, a 30 cm × 5 cm × 5 cm (length × width × height) runway was prepared and covered with white paper. Each mouse was then placed on the runway after its limbs had been dipped in non-toxic dyes of different colors. Of note, the mice were allowed to adapt to the runway for at least 5 min before the formal experiments and outside disturbances were avoided during the whole experiment. The experiments were performed at least three times, and the step distance and stride width were measured and statistically analyzed.

### Determination of the Time of Disease Onset and Survival Curve Analysis

To analyze the time point of disease onset, the body weights of the mice were measured every day. The onset of ALS was recorded as the time point of maximum body weight. Mice that were unable to move from one side to the other in 30 s were considered as dead. The mice were then sacrificed to reduce further pain from respiratory failure according to animal care guidelines. The survival percentages of the mice were statistically analyzed with Kaplan–Meier survival curves.

### Collection of Spinal Cords

Mouse spinal cords were collected from non-transgenic or SOD1^G93A^ mice at the ages of 60 days (in the presymptom stage of ALS), 85 days (in the onset stage of ALS), and 117 days (in the end stage of ALS). In select experiments, mice at the age of 1 month were orally administered rofecoxib (50 mg/kg/day) by gavage until the end stage of ALS. Each mouse was sacrificed by CO_2_ in an enclosed space and then quickly fixed on an operating table. The heart was exposed by opening the chest, and a perfusion needle was inserted into the left ventricle. The perfusion needle was fixed with hemostatic forceps, and the right atrial appendage was cut with scissors. Perfusion was performed with physiological saline and was stopped when the liver became white and clear solution flowed from the right atrium. The spinal cord was collected on ice and separated equally into two parts. One part was submerged in 4% paraformaldehyde; another part was stored at −80°C for protein extraction.

### Western Blot Analysis

Tissues were lysed on ice in RIPA buffer [25 mM Tris–HCl (pH 7.6), 150 mM NaCl, 1% NP-40, 1% sodium deoxycholate, and 0.1% SDS] containing protease inhibitor cocktail (Pierce Chemical Company, Rockford, IL, United States). The total protein concentration was determined with a BCA kit (Pierce Chemical Company, Rockford, IL, United States), and the samples were adjusted to the same concentration. The samples (10–20 μl) were subjected to SDS-PAGE, after which the proteins were transferred to PVDF membranes and probed with a panel of specific antibodies. Each membrane was probed with only one antibody, and antibody binding was visualized by enhanced chemiluminescence (ECL). β-actin was used as a loading control. All of the Western blot experiments were performed in at least triplicate using a different sample preparation each time.

### Tissue Embedding

The spinal cords were immobilized in 4% paraformaldehyde for 48 h, soaked in 70% ethanol for 5 h, and soaked in 80% ethanol overnight at 4 °C. For dehydration, the tissues were soaked sequentially in 90% ethanol for 45 min, 95% ethanol for 30 min (twice), 100% ethanol for 30 min (twice), and 100% ethanol for 45 min. The tissues were then placed in xylene for 20 min and a xylene:soft wax mixture for 45 min before being embedded in soft wax for 50 min and hard wax for 50 min. During the whole process, the organization remained intact; the clearing time was carefully controlled, as an excessive clearing duration can easily disrupt tissue organization. After tissue embedding, serial sections (5 μm thick) were cut using a paraffin slice (Leica, RM2235, Germany), and the sections were used for morphological determination.

### Immunohistochemistry (IHC)

Slides were first dewaxed and rehydrated through a series of xylene and ethanol washes, as follows: 100% xylene for 20 min, 100% ethanol II for 10 min, 100% ethanol I for 10 min, 95% ethanol II for 10 min, 95% ethanol I for 5 min, 90% ethanol for 5 min, 80% ethanol for 5 min, 70% ethanol for 5 min, and deionized water for 5 min. The slides were boiled in a solution of sodium citrate to repair the antigens for 20 min before cooling to room temperature. The tissues were then submerged in 30% hydrogen peroxide to eliminate endogenous peroxidase activity and blocked with goat serum for 45 min. The localization of GFAP (rabbit, 1:400), Iba1 (rabbit, 1:400), NeuN (mouse, 1:400), COX-2 (rabbit, 1:200), IL-1β (rabbit, 1:200), and TNF-α (rabbit, 1:200) was determined using an immunohistochemical staining kit (Fuzhou, Fujian, China) following the manufacturer’s instructions. Briefly, the tissues were incubated with primary antibodies at 4 °C overnight. The next day, the primary antibodies were removed, and the tissues were washed three times with phosphate buffer (PB) for 5 min. The tissues were then incubated with secondary antibodies (goat anti-mouse/rabbit IgG) at room temperature for 1 h and with streptomycin anti-biotin peroxidase for 1 h. After rinsing with PB three times for 5 min, the slides were visualized with DAB, and the reaction was stopped with deionized water. The nuclei of the cells in the spinal cords were stained with hematoxylin for 4 min. The slides were finally dehydrated in 70% ethanol for 5 min, 80% ethanol for 5 min, 90% ethanol for 5 min, 95% ethanol I for 5 min, 95% ethanol II for 10 min, and cleared in 100% xylene for 20 min. After air drying in a fume hood, the slides were mounted with neutral resin before being observed under microscopy. The integrated density of the immunohistochemical staining was measured by ImageJ software.

### Immunofluorescence

To assess colocalization, slices of mouse spinal cords were double-stained with a NeuN antibody (Alexa Fluor 555-labeled secondary IgG) and either an Iba1 or GFAP (Alexa Fluor 488-labeled secondary IgG) antibody. In brief, the spinal cords were submerged in 30% sucrose solution after fixation with 4% paraformaldehyde. Slides (10 μm) were prepared by a cryostat (Leica, CM1850, Shanghai, China) after embedding in optimum cutting temperature (OCT) compound. The tissues were then submerged in 30% hydrogen peroxide to eliminate endogenous peroxidase activity and blocked with goat serum for 45 min. The slides were incubated with antibody specific for GFAP (Rabbit, 1:400) or Iba1 (rabbit, 1:400) mixed with a NeuN antibody (mouse, 1:400) at 4°C overnight. After rinsing with PB three times for 5 min, the slides were incubated with a mixture of fluorescently labeled goat anti-mouse and anti-rabbit secondary antibodies for 1 h. After rinsing with PB, the slides were mounted with fluorescent anti-quenching reagents before being observed under confocal microscopy. The intercellular distance was measured with ImageJ software.

### Nissl Staining

For Nissl staining, the slices were first dewaxed in xylene and rehydrated with gradient alcohol according to the above methods. Then, the samples were soaked in Nissl staining solution at room temperature for 20–30 min. The stained slices were then dehydrated with 90% ethanol, 95% ethanol, 100% ethanol I, and 100% ethanol II for 2 min per wash. Finally, the slices were submerged in xylene for 4 min before being mounted with neutral resin. Nissl staining was performed in the dark.

### Animal Committee

The animal study was reviewed and approved by the Ethics Committees of Capital Medical University and Northeastern University in China.

### Statistical Analysis

All data are represented as the mean ± SE of at least three independent experiments. The statistical significance of differences between means was determined using Student’s *t*-test or one-way analysis of variance, as appropriate. If means were found to be significantly different, multiple pairwise comparisons were performed using Tukey’s *post hoc* test.

## Results

### The Proinflammatory Signaling Cascades of COX-2 Are Activated in Different Stages of ALS

**FIGURE 1:**
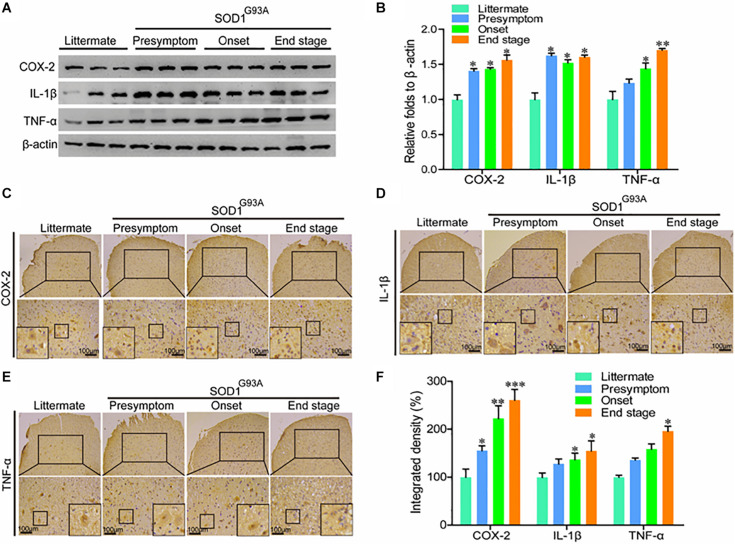
The expression of COX-2, IL-1β, and TNF-α was elevated in the lumbar spinal cords of SOD1^G93A^ mice. **(A,B)** Western blot analysis was used to detect the expression of COX-2, TNF-α, and IL-1β in the spinal cords of SOD1^G93A^ mice at the ages of 60 days (in the presymptom stage of ALS), 85 days (in the onset stage of ALS), and 117 days (in the end stage of ALS). **(C–E)** IHC was used to detect the distribution of COX-2, IL-1β, and TNF-α in the lumbar anterior horn of SOD1^G93A^ transgenic mice at different stages. **(F)** Positive staining for COX-2, IL-1β, and TNF-α in the anterior horns of the spinal cords of SOD1^G93A^ mice was statistically analyzed with Image-Pro Plus (IPP). The data represent the means ± SEs from independent experiments. *p < 0.05; **p < 0.01; ***p < 0.001, compared with non-transgenic mice.

In view of the potential contributions of COX-2 to ALS (Almer et al., 2001), we first determined the protein expression of COX-2 and its downstream signaling targets, IL-1β and TNF-α in SOD1^G93A^ mice of different ages. Western blot analysis demonstrated that the protein expression of COX-2, IL-1β, and TNF-α was markedly elevated in the presymptom, onset, and end stages of ALS (Figures 1A,B). Furthermore, the localization of COX-2, IL-1β, and TNF-α was determined by IHC in the spinal cords of SOD1^G93A^ mice of different ages. The results demonstrated that COX-2 was localized in α motor neurons and glial cells at the early stage of ALS, whereas COX-2 expression in α motor neurons gradually disappeared at the late stage of ALS (Figure 1C). With the loss of motor neurons, large amounts of COX-2-positive smaller cells were observed (Figure 1C), especially at the end stage.

Most proinflammatory cytokines are produced in and secreted by glial cells (Bassett et al., 2012); therefore, we performed immunostaining to detect IL-1β and TNF-α in the spinal cords of SOD1^G93A^ mice. Interestingly, we found that IL-1β and TNF-α, like COX-2, were localized mainly in the α motor neurons and glial cells at the early stage of ALS (Figures 1D,E). At the late stage of ALS, IL-1β, and TNF-α were mainly expressed in smaller cells (Figures 1D,E). However, we do not believe that these proinflammatory cytokines are expressed, produced, and secreted by neuronal cells. The reactive proinflammatory cytokines in neurons might have been recruited from glial cells. Notably, the integrated densities of COX-2, IL-1β, and TNF-α were significantly elevated in the spinal cords of end-stage SOD1^G93A^ mice, even though those proinflammatory factor-expressing motor neurons disappeared (Figure 1F). This observation suggests that the microenvironment in the mouse spinal cord is relatively healthy at the early stage of disease, but that inflammatory factor levels in the anterior horn of the lumbar medulla generally increase gradually with disease progression.

### Glial Cells Are Activated in the Spinal Cords of SOD1^G93A^ Mice

**FIGURE 2:**
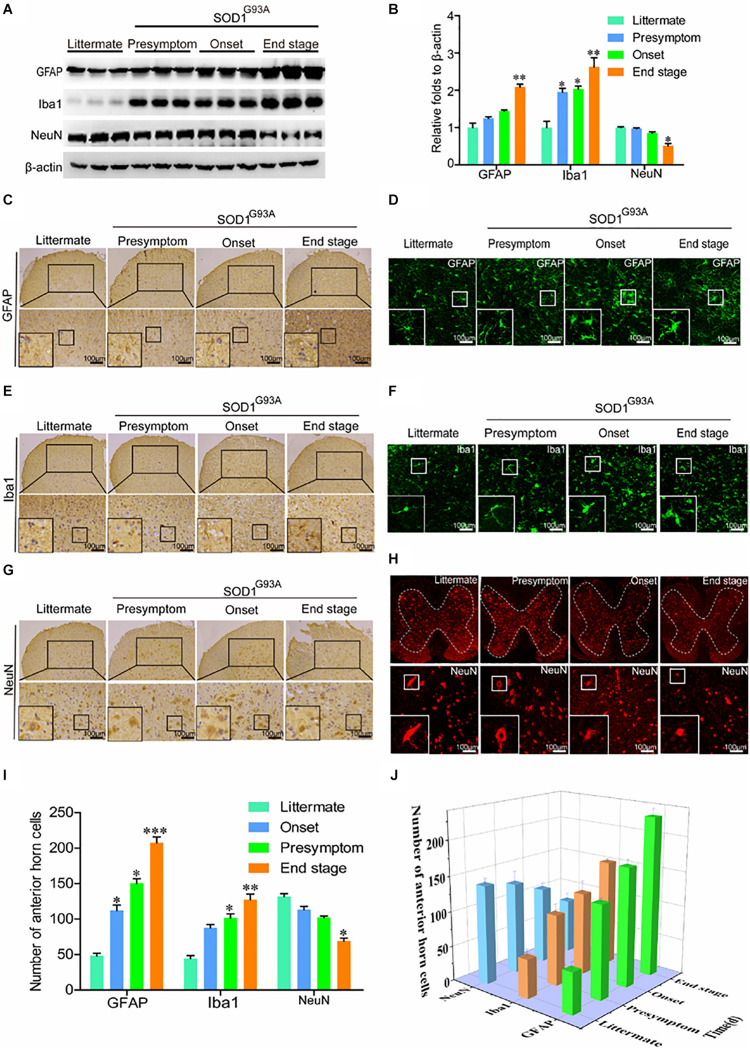
Glial cells were activated, which resulted in loss of motor neurons. **(A,B)** Western blot analysis was employed to detect the protein levels of GFAP, Iba1, and NeuN at the ages of 60 days (in the presymptom stage of ALS), 85 days (in the onset stage of ALS), and 117 days (in the end stage of ALS). The optical densities of GFAP, Iba1, and NeuN were analyzed with ImageJ software. **(C,E,G)** IHC was used to detect the morphology of astrocytes, microglia, and neurons in the lumbar anterior horns of SOD1^G93A^ mice at different stages. **(I)** Positive staining for GFAP, Iba1, and NeuN in the anterior horn of the spinal cord was statistically analyzed with Image-Pro Plus (IPP). **(D,F,H)** Immunofluorescence was used to detect morphological changes in astrocytes, microglia, and neurons via immunostaining for GFAP, Iba1, and NeuN. **(J)** The astrocytes, microglia, and neurons in the lumbar anterior horns of SOD1^G93A^ mice at different stages were counted. The data represent the means ± SEs from independent experiments. *p < 0.05; **p < 0.01; ***p < 0.001, compared with non-transgenic mice.

In view of the above observation, we next determined whether the expression of COX-2, IL-1β, and TNF-α was caused by activation of glial cells. As expected, the protein levels of GFAP and Iba1 were obviously elevated in the spinal cords of SOD1^G93A^ mice (Figures 2A,B). Notably, synthesis of GFAP and Iba1 protein was already stimulated at the presymptom stage of ALS, suggesting the critical roles of neuroinflammation in inducing this disease. Via IHC, we further found that astrocytes and microglia were obviously activated even at the early stage of ALS (Figures 2C,E,G). Morphologically, large numbers of astrocytes proliferated in the spinal cords of SOD1^G93A^ mice with the development and progression of ALS (Figures 2C,D). Interestingly, the body sizes of astrocytes were larger and the processes of astrocytes were longer in SOD1^G93A^ mice at the onset and end stages of disease than in littermate controls and mice in the presymptom stage (Figures 2C,D). In addition, the cell bodies of activated microglia were enlarged, the processes were shortened, and the cells were round, rod-shaped, or ameboid depending on the degree of activation in SOD1^G93A^ mice at the onset and end stage of disease compared with littermate controls and mice in the presymptom stage (Figures 2E,F). Although glial cells were highly activated, neuroinflammation did not affect the protein expression of NeuN at the presymptom stage (Figures 2A,B). However, prolonged activation of neuroinflammation reduced the protein levels of NeuN in the spinal cords of SOD1^G93A^ mice at the end stage (Figures 2A,B). Similarly, the numbers of neurons in the anterior horns were significantly reduced at the end stage of ALS (Figures 2G–I). On the basis of these observations, we conclude that neuroinflammation is positively associated with reductions in motor neuron populations (Figure 2J).

### The Intercellular Distance Between Glial Cells and Neurons in SOD1^G93A^ Mice Becomes Shorter With the Disease Progression

**FIGURE 3:**
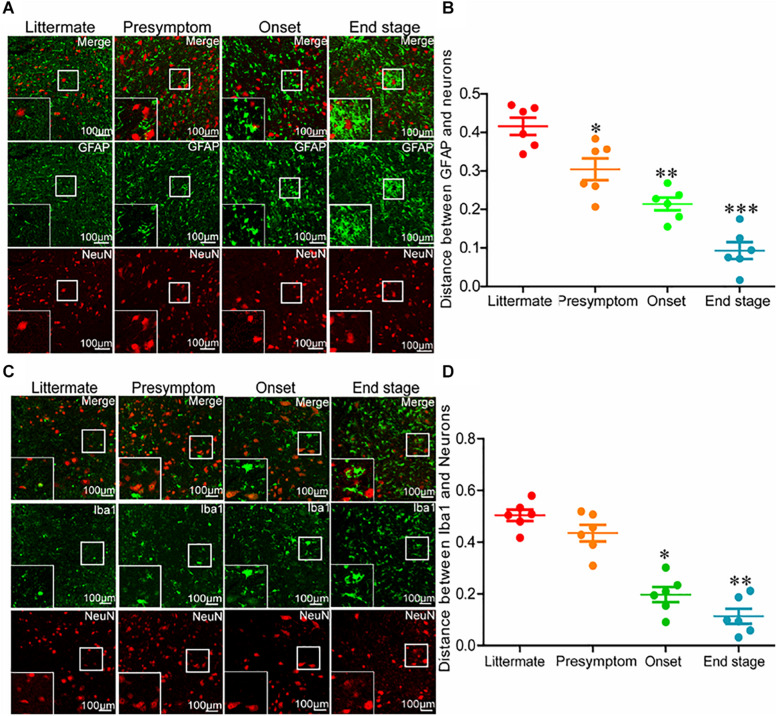
Glial cells moved toward neurons with disease progression of SOD1^G93A^ mice. **(A,B)** The lumbar anterior horns of SOD1^G93A^ mice were double-stained for GFAP (green) and NeuN (red). The distance between astrocytes and neurons was calculated and analyzed with ImageJ software. **(C,D)** The lumbar anterior horns of SOD1^G93A^ mice were double-stained for Iba1 (green) and NeuN (red). The distance between microglia and neurons was calculated and analyzed with ImageJ software. The data represent the means ± SEs from independent experiments. *p < 0.05; **p < 0.01; ***p < 0.001, compared with non-transgenic mice.

Even though neuroinflammation caused the death of motor neurons, why the neuroinflammatory reaction significantly elevated as early as in the presymptom stage could not induce neuronal death at the early stage of ALS remained unclear. Thus, we investigated the relationships between glial cells and neurons at different stages of ALS. For this purpose, the spinal cords of SOD1^G93A^ mice were double-stained for GFAP and NeuN. Astrocytes were more closely associated with neurons at the end stage of ALS than that at the early stage (Figures 3A,B). In other words, the intercellular distance between astrocytes and neurons in SOD1^G93A^ mice became shorter with disease progression. On the basis of this observation, we further sought to investigate whether this was also the case for microglia and neurons. To this end, we double-stained the spinal cords for Iba1 and NeuN. Consistent with the previous findings, microglia were more closely associated with neurons at the late stage of ALS than that at the early stage (Figures 3C,D). These observations suggest that glial cells might be the mediators of motor neuron death.

### Rofecoxib Treatment Increases the Intercellular Distance Between Glial Cells and Neurons at the Late Stage of ALS

**FIGURE 4:**
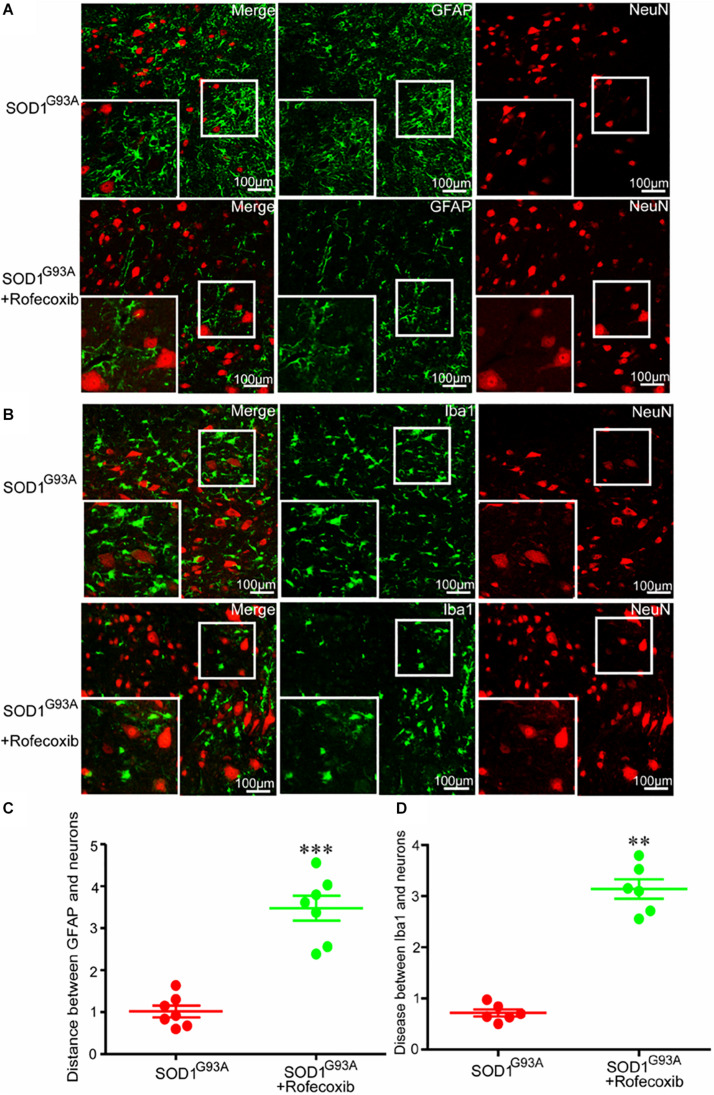
Rofecoxib treatment increased the distance between glial cells and neurons in SOD1^G93A^ mice. **(A,B)** The spinal cords were collected for double immunofluorescence staining. **(C,D)** The distance between glial cells and neurons was calculated and analyzed by ImageJ software. The data represent the means ± SEs from independent experiments. **p < 0.01; ***p < 0.001, compared with vehicle-treated SOD1^G93A^ mice.

Given the above findings, we next investigated whether the close relationship of glial cells and neurons were caused by overexpression of COX-2 in SOD1^G93A^ mice. We first orally administered rofecoxib (50 mg/kg/day), a specific inhibitor of COX-2, to mice according to the methods in a previous study (Klivenyi et al., 2003). Via double staining, we measured the intercellular distance between glial cells and neurons in the spinal cords of SOD1^G93A^ mice. As expected, we found that rofecoxib treatment clearly increased the intercellular distances both between astrocytes and neurons, and microglia and neurons at the late stage of ALS (Figure 4). This observation indicates that the specific COX-2 inhibitor rofecoxib might be a promising agent for ALS treatment.

### Rofecoxib Treatment Partially Prevents the Loss of Motor Neurons by Deactivating Glial Cells

**FIGURE 5:**
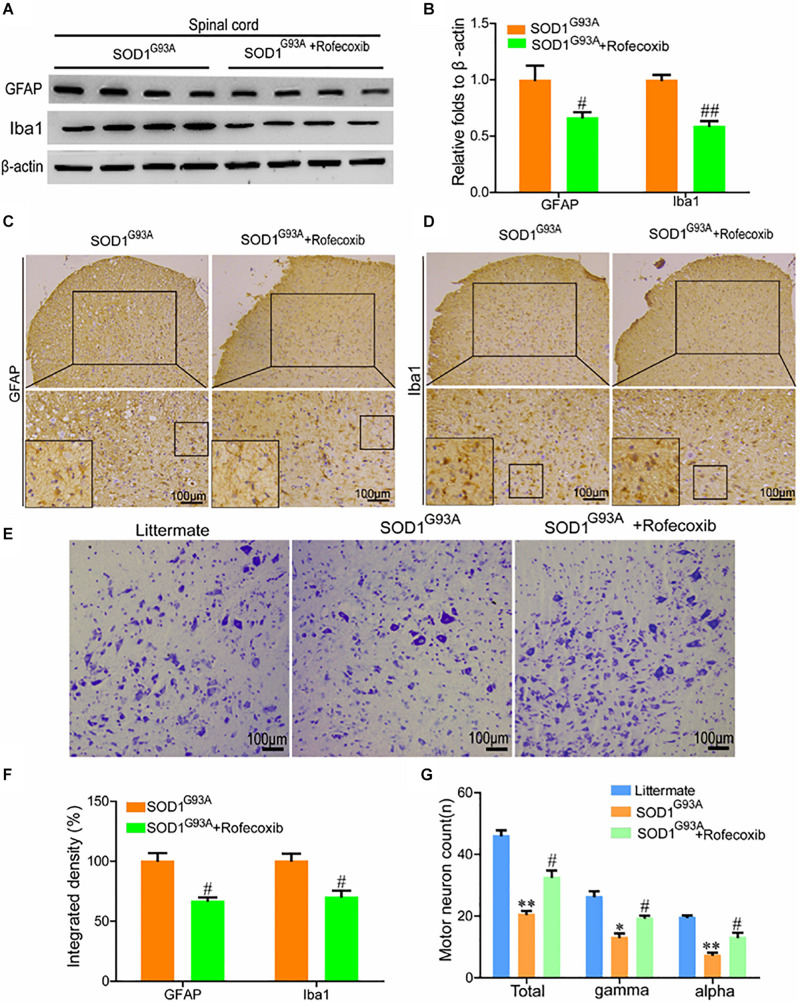
Rofecoxib treatment decreased the activity of glial cells, which resulted in increased numbers of motor neurons. **(A,B)** Western blot analysis was employed to detect the protein levels of GFAP and Iba1. The optical densities of GFAP and Iba1 were analyzed with ImageJ software. **(C,D,F)** IHC was used to detect the morphology and integrated density of astrocytes and microglia in the lumbar anterior horns of SOD1^G93A^ mice. **(E,G)** Spinal cords were collected for Nissl staining. The numbers of motor neurons were calculated and analyzed by Image-Pro Plus (IPP). The data represent the means ± SEs from independent experiments. *p < 0.05; **p < 0.01, compared with non-transgenic mice. ^#^*p* < 0.05, ^##^*p* < 0.01, compared with vehicle-treated SOD1^G93A^ mice.

To further validate the above observations, the spinal cords of SOD1^G93A^ mice were examined for GFAP and Iba1 expression. The results of Western blot analysis demonstrated that rofecoxib treatment reduced the protein levels of GFAP and Iba1 in the spinal cords of SOD1^G93A^ mice (Figures 5A,B). Via morphological analysis, we further found that rofecoxib deactivated astrocytes by decreasing astrocyte cell body sizes and shortening astrocyte processes (Figure 5C). With regard to microglia, the populations of round and rod-shaped cells and the process densities of cells were markedly decreased after treatment with rofecoxib (Figure 5D). Moreover, the integrated densities of GFAP and Iba1 in astrocytes and microglia were also lower in rofecoxib-treated SOD1^G93A^ mice than in untreated controls (Figure 5F). Since neuroinflammation was found to be attenuated, the effects on motor neurons were further investigated. Nissl staining revealed that the numbers of motor neurons were obviously reduced in the spinal cords (Figures 5E,G). In more detail, the α and γ motor neuron numbers were significantly lower in SOD1^G93A^ mice than in non-transgenic controls (Figures 5E,G). Rofecoxib treatment clearly restored the numbers of total motor neurons, including α and γ motor neurons (Figures 5E,G). These results reinforce the notion that rofecoxib treatment prevents loss of motor neurons by deactivating neuroinflammation, suggesting that COX-2 plays critical roles in ALS aggravation by inducing inflammation.

### Rofecoxib Treatment Suppresses Proinflammatory Cytokine Expression in the Lumbar Spinal Cords of SOD1^G93A^ Mice

**FIGURE 6:**
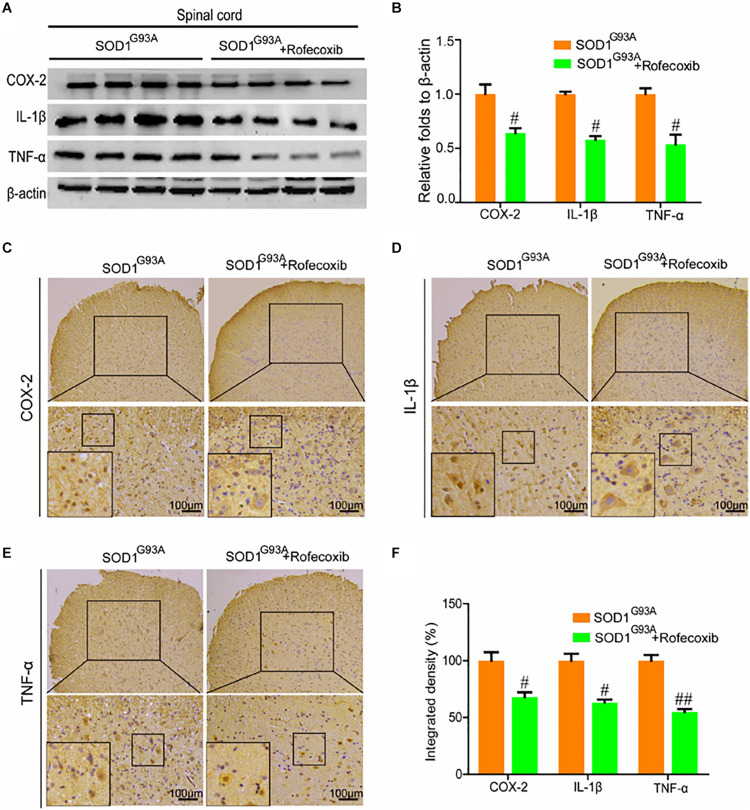
Rofecoxib treatment decreased the expression of COX-2, IL-1β, and TNF-α in the spinal cords of SOD1^G93A^ mice. **(A,B)** Total protein was extracted and subjected to Western blot analysis with antibodies specific for COX-2, IL-1β, and TNF-α. The optical densities of the bands were calculated and analyzed with ImageJ software. **(C,D,E)** IHC was employed to determine the distributions of the COX-2, IL-1β, and TNF-α-positive cells in the lumbar anterior horns of SOD1^G93A^ mice. **(F)** Positive staining for COX-2, IL-1β, and TNF-α was assessed and analyzed with Image-Pro Plus (IPP). The data represent the means ± SEs from independent experiments. ^#^*p* < 0.05; ^##^*p* < 0.01, compared with vehicle-treated SOD1^G93A^ mice.

Given the ability of rofecoxib to deactivate glial cells, we further determined whether rofecoxib could suppress the expression of proinflammatory cytokines by inhibiting COX-2. To this end, Western blot analysis was carried out to measure the expression of COX-2, IL-1β, and TNF-α in the spinal cords of SOD1^G93A^ mice. The results demonstrated that rofecoxib treatment clearly inhibited the expression of IL-1β and TNF-α by decreasing the protein expression of COX-2 in the spinal cords of SOD1^G93A^ mice (Figures 6A,B). Interestingly, the morphological analysis revealed that COX-2 was redistributed to the surviving large motor neurons (Figure 6C). As the downstream targets of COX-2, IL-1β was also relocated to the surviving large motor neurons after treatment with rofecoxib (Figure 6D). In contrast, TNF-α was not obviously redistributed to the surviving large motor neurons after treatment with rofecoxib (Figure 6E). However, the integrated densities were reduced in the rofecoxib-treated groups (Figure 6F).

### Rofecoxib Slows the Disease Progression of SOD1^G93A^ Mice

**FIGURE 7:**
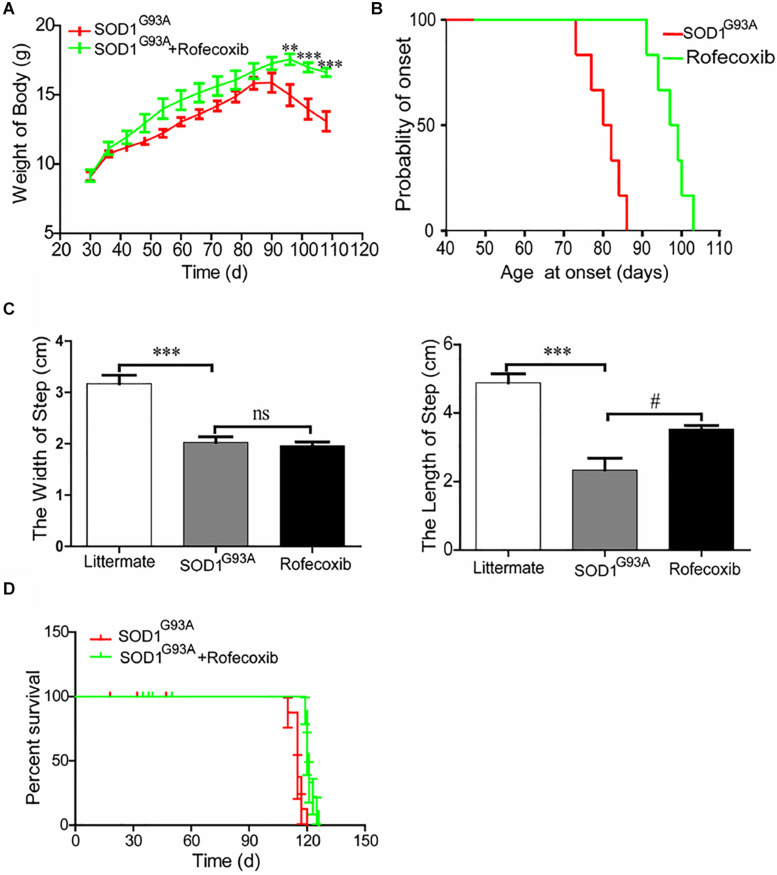
Rofecoxib treatment alleviated the disease progression of SOD1^G93A^ mice. SOD1^G93A^ mice were orally administered rofecoxib (50 mg/kg/day) until dead or the end stage of ALS. **(A)** Body weight was monitored over the entire lifespans of SOD1^G93A^ mice treated or not treated with rofecoxib (50 mg/kg/day). **(B)** The probability of ALS onset was determined on the basis of body weight. **(C)** The step size and stride width were further calculated and statistically analyzed with GraphPad Prism 5. **(D)** The survival percentages of the different groups of mice were calculated. The data represent the means ± SEs from independent experiments. For body weight, **p < 0.01; ***p < 0.001, compared with vehicle-treated SOD1^G93A^ mice. For the step size and stride width, ***p < 0.001, compared with non-transgenic mice and ^#^*p* < 0.05, compared with vehicle-treated SOD1^G93A^ mice.

To further assess the effects of rofecoxib on the disease progression of SOD1^G93A^ mice, the body weights of mice were monitored every day. The results demonstrated that the body weights of SOD1^G93A^ mice significantly decreased with disease progression, especially in vehicle-treated controls (Figure 7A). The average body weight of rofecoxib-treated SOD1^G93A^ mice was higher than that of vehicle-treated SOD1^G93A^ mice (Figure 7A). Using these data, we further analyze the onset time of ALS. The average onset time was obviously postponed in rofecoxib-treated SOD1^G93A^ mice than in vehicle-treated controls (Figure 7B).

Gait analysis was also carried out on SOD1^G93A^ mice with or without rofecoxib treatment. The results demonstrated that rofecoxib-treated mice showed better locomotion ability than vehicle-treated mice. We further found that rofecoxib treatment increased the step length of SOD1^G93A^ mice without considerably changing the stride width (Figure 7C). Given this observation, we hypothesized that rofecoxib increases the possibility of survival in SOD1^G93A^ mice. To test this hypothesis, we determined the survival time of mice with or without rofecoxib treatment. The data revealed that rofecoxib treatment modestly prolonged the survival times of SOD1^G93A^ mice (Figure 7D). Taken together, our findings reveal that rofecoxib shows neuroprotective effects by targeting COX-2 proinflammatory signaling cascades in ALS mice.

## Discussion

The expression of COX-2 is tightly regulated under physiological conditions. However, during the course of ALS development and progression, the activity of COX-2 is markedly activated in the spinal cord (Yiangou et al., 2006). In addition, COX-2 expression is restricted to neurons under physiological conditions, but in the context of ALS, the immune activity of COX-2 in the spinal cord spreads to activated glial cells (Yiangou et al., 2006). Although COX-2 is expressed in cells other than glial cells (Maihofner et al., 2003), high expression of COX-2 in glial cells likely promotes the development of ALS through an inflammatory mechanism (Almer et al., 2001). Consistent with this possibility, PGE_2_, an important metabolic product of COX-2, is reported to be critical for inducing inflammation during the course of ALS development and progression (Almer et al., 2002). In addition, rofecoxib treatment significantly reduces the levels of PGE_2_ in the spinal cords of SOD1^G93A^ mice, which improves motor performance, attenuated weight loss, and extends survival (Klivenyi et al., 2003). In particular, the expression of COX-2 is stimulated in the anterior horns of the spinal cords of SOD1^G93A^ mice throughout the whole process of ALS, which coincides with the death of motor neurons (Kiaei et al., 2005). Consistent with these observations, we found that COX-2 expression was already stimulated as early as in the presymptom stage and that it remained at high levels until the end stage of disease (Figure 1A). Although the expression of COX-2 was consistently induced during the course of ALS development and progression, morphological analysis further revealed that its expression shifted from the neurons in the early stage to glial cells in the later stage (Figure 1C). This observation indicates the effects of neuroinflammation on neuronal death and gliosis.

Apart from COX-2, which was overactivated, the downstream COX-2 targets, IL-1β and TNF-α, were also activated in SOD1^G93A^ mice at the presymptom stage (Figures 1A,D,E). In agreement with our observations, mutant (m) SOD1 has been reported to induce the expression of IL-1β, which accelerates the pathogenesis of ALS (Meissner et al., 2010). In addition, treatment with Kineret (anakinra), an antagonist of the IL-1β receptor, has been found to prolong the survival of ALS mice by improving the biological functions of motor neurons (Martz, 2010). Plasma TNF-α levels have also been found to be elevated in sALS patients compared to normal controls (Cereda et al., 2008). Notably, iron has been found to be critical for inducing the secretion of TNF-α in SOD1^G93A^ mice (Lee et al., 2015). On the basis of these observations, we extended previous research by testing the effects of rofecoxib, a COX-2-specific inhibitor. In view of the early activation of neuroinflammation, we started to treat SOD1^G93A^ mice at the age of 1 month. Similar to our study, several previous studies have implemented early treatment of SOD1^G93A^ mice with various drugs; for example, in one study, mice were treated with lysine acetylsalicylate, a soluble salt of aspirin (Barneoud and Curet, 1999). More similarly, in another study, SOD1^G93A^ mice were treated with rofecoxib at the age of 30 days (Klivenyi et al., 2003). Early administration might be the reason why most drugs show efficacy in animal models of ALS, but not in clinical trials, as drugs are often administered to humans late after the appearance of symptoms (Petra Kaufmann et al., 2009; Stommel et al., 2009; Miller et al., 2011). In this study, treatment with rofecoxib suppressed the expression of IL-1β and TNF-α in SOD1^G93A^ mice (Figure 6). Although there has been no direct evidence that rofecoxib inhibits the expression of TNF-α in SOD1^G93A^ mice, rofecoxib has been shown to inhibit TNF-α expression in RAW264.7 cells (Babcock et al., 2004). In addition, rofecoxib has been shown to suppress acute inflammation in carrageenan-activated rats by inhibiting the production of TNF-α and IL-1β (Pinheiro and Calixto, 2002). These findings suggest that COX-2 is a pivotal molecule regulating neuroinflammation, which is responsible for the development and progression of ALS.

We continued to elucidate the effects of proinflammatory cytokines on the fate of motor neurons. Overload of proinflammatory cytokines resulted in motor neuron loss (Figures 2A,B,G–I). Consistent with our observations, IL-1β has been found to increase necrotic neuronal death in the developing rat hippocampus by activating the type I IL-1 receptor (Medel-Matus et al., 2014). In addition, IL-1β induces excitotoxic motor neuron injury in the spinal cord during acute viral encephalomyelitis (Prow and Irani, 2008). Apart from IL-1β, TNF-α has been suggested to induce the death of motor neurons by activating NF-κB in the spinal cords of rats (Tolosa et al., 2011). Notably, a lack of type II TNF-α receptor protects the motor neurons from death in SOD1^G93A^ mice (Tortarolo et al., 2015).

Interestingly, activated microglial cells (BV-2 cells) have been found to facilitate the death of motor neurons by activating TNF-α (He et al., 2002). This observation prompted us to investigate the relationship between glial cells and neurons. Our results demonstrated that glial cells moved close to neurons during the development and progression of ALS (Figure 3). These findings supported the observations that the protein levels of NeuN appeared to be decreased only at the end stage of ALS (Figures 2A,B). In addition, these findings indicate that activated glial cells can induce motor neuronal death only when the motor neurons are within a certain distance.

Given the pivotal roles of neuroinflammation in ALS, we sought to investigate whether the disease can be alleviated by inhibiting proinflammatory signaling cascades. Since we identified COX-2 as a regulator of neuroinflammation, we further treated SOD1^G93A^ mice with rofecoxib. The results clearly demonstrated that rofecoxib deactivated glial cells by suppressing the expression of IL-1β and TNF-α in the spinal cords of SOD1^G93A^ mice (Figures 5, 6). As an inhibitor of COX-2, rofecoxib has been reported to be responsible for attenuating the activity of glial cells (Scali et al., 2003). In SOD1^G93A^ mice, rofecoxib (10 mg/kg) treatment at postnatal day 60 has been found to obviously delay the onset of motor impairment (Azari et al., 2005). However, a dose of 10 mg/kg has not been found to affect the survival of ALS mice (Azari et al., 2005). We thereby increased the dose to 50 mg/kg. Although there is no direct evidence regarding the best dose for ALS treatment, rofecoxib shows relatively higher gastrointestinal safety than other COX-2-specific inhibitors, such as naproxen (Bombardier et al., 2000). In addition, high-dose rofecoxib treatment can suppress the activity of NF-κB, which mediates the expression of proinflammatory genes in a series of stimulating environments (Niederberger et al., 2002). Thus, we selected a relatively high dose of rofecoxib for SOD1^G93A^ mouse treatment. Even though 50 mg/kg of rofecoxib treatment alleviated the loss of motor neurons by deactivating glial cells and delayed disease onset, it only weakly prolonged the survival of mice (Figure 7D). There may be several reasons for this weak survival-promoting effect. First, the neuroinflammatory process in ALS is quite complex; early treatment targeting COX-2 may exert more effective protection in the presymptom stage. Erythropoietin significantly downregulates the production of proinflammatory cytokines, such as IFN-γ, TNF-α, IL-1β, CCL2, CCL5, CXCL10, and IL-17A; upregulates the expression of anti-inflammatory cytokines, such as IL-4, IL-10, and TGF-β; and prevents the motor neuron cell death. However, erythropoietin only mildly delays the onset of ALS mice (Noh et al., 2014). Second, similar to rofecoxib in the current study, the anti-inflammatory drugs, such as D-penicillamine, a copper chelator, and sulindac, a COX-2 inhibitor, can only extend the survival time by about 10% (Hottinger et al., 1997; Kiaei et al., 2005). Like rofecoxib, several other anti-neuroinflammatory interventions have also yielded better effects in delaying disease onset than in prolonging survival in SOD1^G93A^ mice, such as cromolyn sodium (Granucci et al., 2019), tempol (Chiarotto et al., 2019), and lysine acetylsalicylate (Barneoud and Curet, 1999). Third, many other mechanisms besides neuroinflammation have been confirmed to be related to the pathogenesis of ALS, such as protein aggregation (Brown and Al-Chalabi, 2017), oxidative stress (Bozzo et al., 2017), glutamate excitotoxicity (Blasco et al., 2014), autophagy abnormality (Nassif and Hetz, 2011), mitochondrial structure and function abnormality (Golpich et al., 2016), and endoplasmic reticulum stress (Matus et al., 2013). Therefore, modestly prolonging the survival of SOD1^G93A^ mice by rofecoxib is accepted by only inhibiting neuroinflammation.

As the above discussion, more than 30 different mutated pathological genes have been found to participate in regulating ALS over the past 20 years. However, SOD1^G93A^ mouse is the best experimental model for ALS at present, even though it could not thoroughly mimic the chronic disease process of ALS. In this kind of experimental model, rofecoxib probably shows modest effects because of high transgene copy number in mice compared to that of human patients. On the other hand, rofecoxib might not significantly prolong the survival of SOD1^G93A^ mice to a large extent if the drug exerts its effects via other genes, such as tdp43, Fus/TLS, c9orf72, etc. Therefore, establishing a better experimental model of ALS with more chronic disease process like human patients will be useful for testing the clinical translation of potential drug candidates. However, the current study is just aiming to reveal the inherent mechanisms of COX-2 in aggravating the progression of ALS; SOD1^G93A^ mouse is still the best experimental model for ALS, and it is understandable that rofecoxib shows relative modest effects on prolonging the survival of SOD1^G93A^ mice.

Moreover, COX-2 is expressed not only in the central nervous system, such as the neurons of forebrain, cortex, and hippocampus (Kaufmann et al., 1996), but also in human tracheal epithelial cells (Walenga et al., 1996) and the adjacent epithelial cells in the macula densa of the rat kidney (Harris et al., 1994). In human monocytes, mouse NIH3T3, and human umbilical vein endothelial cells, COX-2 is expressed and distributed on the endoplasmic reticulum and nuclear membrane (Spencer et al., 1998). In various studies, COX-2 was reported to be an inducible enzyme for inflammation (Gilroy and Colville-Nash, 2000). Specifically, mast cells have been suggested as an important cellular site of COX-2 activity during acute peritoneal inflammation (Elzbieta et al., 2009). COX-2 activation is also responsible for the delayed phase of PGD_2_ synthesis in mast cells (Reddy et al., 1999). Furthermore, COX-2 is predominantly expressed and activated at the peak of inflammation (Kolaczkowska et al., 2009). Moreover, they also recognized tissue-resident peritoneal leukocytes, especially macrophages and mast cells as the important cellular sites for activating COX-1 and COX-2 in all stages of acute peritoneal inflammation (Kolaczkowska et al., 2009). By inhibiting the expression of COX-2 and the production of TNF-α, IL-6, and IL-8, transgenic panax ginseng shows its therapeutic effects on mast cell-mediated inflammatory diseases (Kim et al., 2007). More closely, COX-2 specific inhibitor, including meloxicam and NS-398, showed their effects on suppressing the release of histamine (Grosman, 2007), which is associated with mast cell-mediated allergic inflammation (Singh et al., 2012). All these evidence confirmed the pivotal roles of COX-2 in peripheral inflammation, leading to the onset of diseases.

COX-2-specific inhibitors, including rofecoxib, celecoxib, and parecoxib, have shown their effects on anti-IgE-induced histamine release from human skin mast cells and basophils (Gibbs and Boehncke, 2003). More specifically, the inhibitory effects of endocannabinoid 2-arachidonylglycerol on the immunological activation of guinea pig mast cells were blocked by the treatment with rofecoxib in nitric oxide (NO)- and eicosanoid-producing mechanisms (Vannacci et al., 2004). Although there is no direct evidence suggesting the efficacy of rofecoxib on treating ALS via inhibiting peripheral inflammation, activated macrophages, mast cells, and T cells have been confirmed to be responsible for inducing the inflammation in the spinal cords of ALS (Graves et al., 2004). Consistently, dysregulation of immune system has been reported in the spinal cord of SOD1^G93A^ mice (Figueroa-Romero et al., 2019). By the involvement of immune system in the pathogenesis of ALS, stimulating the protective effects of the immune system in humans, including intravenous immunoglobulins, and other experimental interventions such as antibodies, vaccination, minocycline, and neural stem cells have shown the promise efficacy on treating animal models of ALS (Chio et al., 2010). Given the effects of rofecoxib on immune system (Walmesley et al., 2007; Zhang and Zhou, 2010), it is reasonable to believe that rofecoxib probably exerts its effects on treating ALS via modulating the activity of immune systems.

Thus, we speculate that rofecoxib provides neuroprotective effects by regulating the inflammatory response, which is too excessively activated to be effectively regulated after disease onset. Similar to the case in human ALS, pathological changes are particularly obvious in SOD1^G93A^ mice when symptoms begin to be apparent. The ALS drugs approved by the FDA thus far show only rather weak survival-prolonging effects and do not prevent or reverse the progression of ALS (Miller et al., 2000). Therefore, rofecoxib might be a good preventive drug for treating ALS and we still have a long way to go in developing treatments that prolong the survival of patients with ALS.

## Data Availability Statement

The raw data supporting the conclusions of this article will be made available by the authors, without undue reservation, to any qualified researcher.

## Ethics Statement

The animal study was reviewed and approved by the Ethics Committees of Xuanwu Hospital, Capital Medical University, and Northeastern University.

## Author Contributions

Y-HZ and P-PG conserved and performed all of the experiments, participated in the design of the study, and wrote the manuscript. S-QZ carried out select experiments. PW (along with Y-SG) interpreted the data and wrote the manuscript. All authors contributed to the article and approved the submitted version.

## Conflict of Interest

The authors declare that the research was conducted in the absence of any commercial or financial relationships that could be construed as a potential conflict of interest.
